# Causal relationship between hypertension and epilepsy: a mendelian randomization study

**DOI:** 10.1186/s42494-024-00152-9

**Published:** 2024-03-08

**Authors:** Zhen Sun, Tong Jiang, Mengwen Zhang, Yulong Li, Jing Zhang, Yanping Sun, Xiaofeng Yu

**Affiliations:** 1 Department of Neurology, The Affiliated Hospital of Qingdao University, Qingdao, 266000 China; 2 Department of Endocrinology and Metabolism, The Affiliated Hospital of Qingdao University, Qingdao, 266000 China; 3 Department of Neurology, Qingdao Eighth People’s Hospital, Qingdao, 266000 China

**Keywords:** Mendelian randomization, Epilepsy, Hypertension, Systolic blood pressure

## Abstract

**Background:**

Recent studies suggest that hypertension may increase the risk of epilepsy onset, revealing intricate interactions between cardiovascular health and neurological disorders, thus emphasizing the significance of conducting further investigations into their connection. This study aimed to investigate the potential causality between hypertension, either in systolic or diastolic blood pressure, and epilepsy, using a Mendelian randomization strategy.

**Methods:**

A two-sample Mendelian randomization design was used in this study. We extracted data from the UK Biobank, FinnGen, and the International Consortium of Blood Pressure, utilizing blood pressure-related single nucleotide polymorphisms as instrumental variables to evaluate the influence of hypertension on the risk of epilepsy. Inverse variance weighted, weighted median, and MR-Egger approaches were used for analysis.

**Results:**

There was a potential association between hypertension, primarily in systolic blood pressure, and an elevated epilepsy risk, while the relationship between hypertension in diastolic blood pressure and epilepsy risk remained inconclusive. Sensitivity analyses suggest an absence of substantial heterogeneity and confounding effects, suggesting the reliability of our findings.

**Conclusions:**

Our study lays the groundwork for further investigations into the mechanisms of this causal relationship, which may potentially involve vascular change, neuroinflammatory pathways, and alterations in cerebral blood flow, which are crucial for understanding the complex hypertension-epilepsy nexus.

## Background

Epilepsy is a multifaceted neurological disorder characterized by abrupt aberrant neuronal discharges within the brain, leading to transient cerebral dysfunction [1]. It has a global incidence of 50 per 100,000 individuals, affecting an estimated total number of 70 million individuals. Epilepsy is defined as two unprovoked seizures occurring more than 24 h apart, a single unprovoked seizure with a high risk of recurrence (i.e., > 60% over the next 10 years), or the diagnosis of an epilepsy syndrome. The etiology of epilepsy is multifactorial, encompassing genetic, structural, metabolic, infectious, and immune factors. Despite the significant advancements in research methodologies, the causative factors and the underlying pathologies remain elusive in approximately 50% of newly diagnosed epilepsy cases [2, 3].

A meta-analysis revealed a median weighted standardized mortality ratio of 2.3 in epilepsy patients across all age groups compared to the general population, with a higher mortality ratio observed in pediatric patients. Therefore, premature mortality in epilepsy patients is a significant public health concern, underscoring the importance of early screening and prevention [4]. The burden of epilepsy is especially huge in low- and middle-income countries, with profound implications for the affected individuals, particularly children and adolescents, including educational disruption and societal stigma. People with epilepsy are more likely to have mental health disorders, which further diminish their quality of life. Due to the resource constraints and societal stigma, a significant proportion of patients are unable to receive appropriate treatment [5]. Therefore, identifying the potential risk factors for epilepsy is of great importance to reduce morbidity and disability, alleviate the economic burden, and enhance the quality of life of patients.

Hypertension serves as a significant precipitant of cardiovascular diseases, premature death, chronic kidney disease, and dementia [6, 7]. The global prevalence of hypertension is increasing. In 2015, the number of deaths associated with hypertension exceeded 8 million, with the vast majority (88%) occurring in low- and middle-income countries [7]. Numerous studies and case reports have documented the co-occurrence of epilepsy and hypertension. Some studies have proposed that hypertension serves as an independent predictor for late-onset epilepsy, irrespective of the vascular injury [8]. However, the causal relationship between the two conditions remains ambiguous. The renin-angiotensin system (RAS) may be involved in the direct relationship between hypertension and epilepsy. Conversely, the association of hypertension with epilepsy could also be attributed to its role in exacerbating cerebrovascular disease [9, 10]. Understanding the relationship between epilepsy and hypertension holds promise for mitigating the risk of epilepsy and reducing the recurrence rate in patients.

Randomized controlled trials (RCTs) are widely recognized as the "gold standard" for medical research to establish a causality. The strength of RCTs lies in the randomized design, which effectively balances both known and unknown confounding factors, thereby reducing the bias and enhancing the reliability of causal inference. However, RCTs also encounter significant limitations when exploring the relationship between two diseases. Ethical considerations often render many RCTs unfeasible. For instance, it is not ethically permissible to intentionally inflict a disease on a population to ascertain whether it escalates the risk of another disease. The Mendelian randomization (MR) method offers a compelling alternative. MR in genetic epidemiology uses genetic variation closely associated with an exposure factor as an instrumental variable. This approach estimates the causal impact of suspected exposures on outcomes. When the fundamental assumptions are satisfied, MR effectively mitigates the inherent limitations of traditional observational studies, such as confounding and reverse causality. This is attributed to the use of genetic alleles in the MR method, which are randomly assigned to offspring prior to birth. Consequently, MR is advantageous in eliminating confounding factors and identifying causal determinants of outcomes. Therefore, MR analysis can circumvent many potential methodological limitations inherent in observational studies, such as reverse causality bias and confounding. MR is a potent tool for unveiling causal relationships between exposures and outcomes [11–13].

Considering the global burdens of epilepsy and hypertension, deciphering the potential causal interplay between these two conditions is of significant public health importance. MR studies on the causal nexus between hypertension and epilepsy can not only advance our understanding of the risk factors of epilepsy, but also have practical implications for clinical management of epilepsy.

## Methods

This study employed a two-sample MR approach to examine the influence of hypertension (systolic blood pressure ≥ 140 mmHg or diastolic blood pressure ≥ 90 mmHg) on epilepsy.

**Fig. 1:**
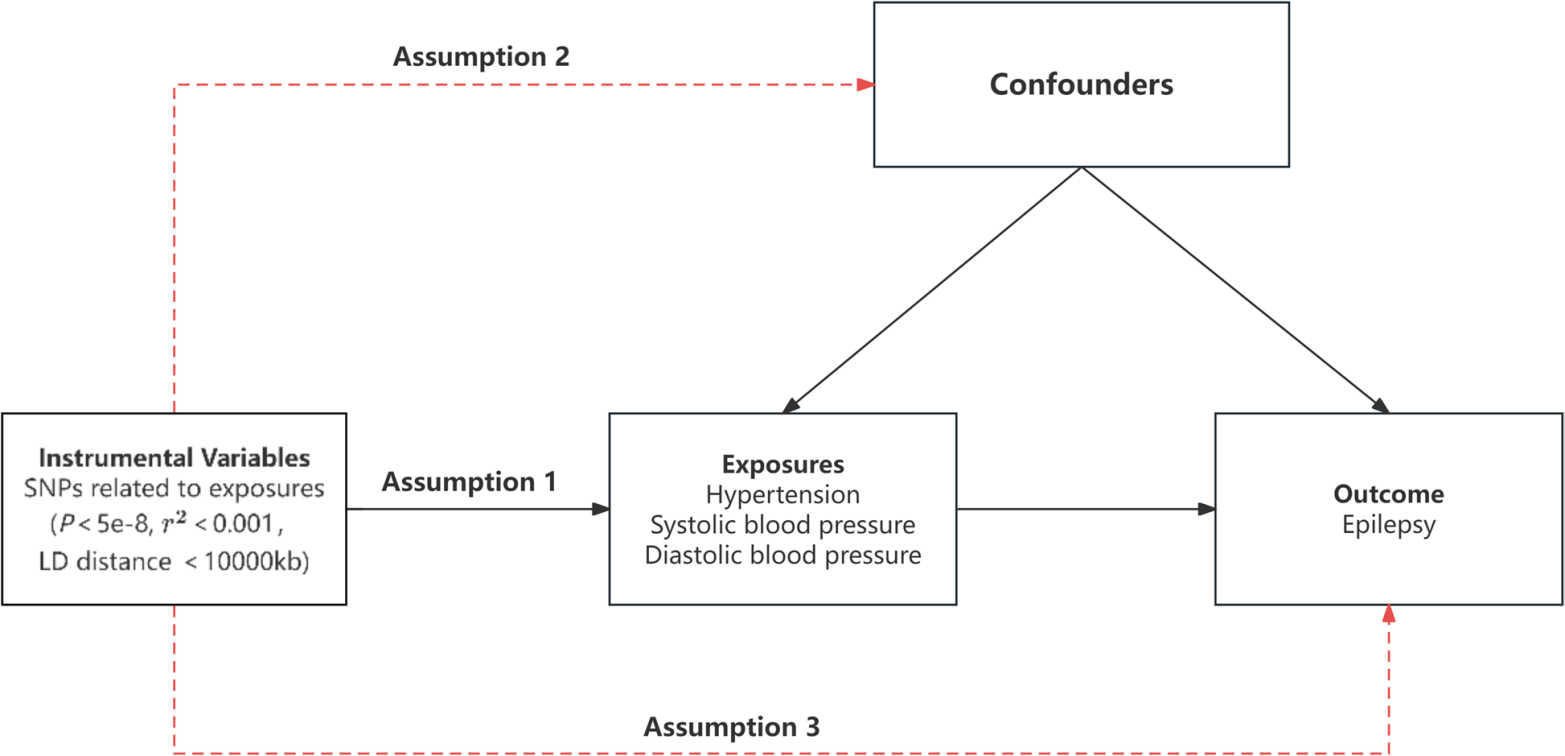
The three primary assumptions of a two-sample Mendelian randomization study: the red dashed lines represent that the genetic variants are independent of confounding factors and the outcome. SNPs, single nucleotide polymorphisms

Single nucleotide polymorphisms (SNPs) associated with blood pressure were selected as instrumental variables based on three core assumptions: (1) the chosen SNP must exhibit a correlation with the exposure variable, i.e., the presence or variation of the SNP can modulate the level of the exposure variable; (2) the selected SNP should be independent of any confounding variables that could potentially obscure the relationship between exposure and outcome variables, i.e., the distribution of SNPs remains unaffected by these confounding variables; and (3) the influence of the selected SNP on the outcome variables should be mediated solely through its impact on the exposure variables, without any direct effect on the outcome variables or any influence via alternative pathways (Fig. 1).

As depicted in Table 1, our data repositories were sourced from the UK Biobank [14], FinnGen [15], and the International Consortium of Blood Pressure. Hypertension data were from FinnGen, encompassing a European population of both sexes, including 42,857 hypertension patients and 175,935 healthy controls. Data of systolic and diastolic blood pressure were derived from the International Consortium of Blood Pressure, again representing a European population of both sexes, with a total of 757,601 samples. To mitigate the potential bias introduced by racial disparities, we refrained from selecting data from the International Anti-epilepsy Society when choosing the epilepsy data source. Given the mixed population of this database, we opted for the UK Biobank, which represents a European population. This database includes 3810 epilepsy patients and 459,123 healthy controls, with both sexes represented.

**Table 1:** Detailed information of studies and datasets used for analyses

Phenotype	GWAS ID	Population	Sex	*N* of cases	*N* of controls	Number of SNPs	Sample size
Epilepsy	ukb-b-16309	European	Males and Females	3810	459,123	9,851,867	462,933
Hypertension	finn-b-I9_HYPTENSESS_EXNONE	European	Males and Females	42,857	175,935	16,380,466	218,792
Systolic blood pressure	ieu-b-38	European	Males and Females	NA	NA	7,088,083	757,601
Diastolic blood pressure	ieu-b-39	European	Males and Females	NA	NA	7,160,619	757,601

### Instrumental variable selection

Blood pressure-associated SNPs were selected as instrumental variables, based on a strict genome-wide significance threshold (*P* < 5e-8). Furthermore, SNPs exhibiting linkage disequilibrium (LD;* r*^2^ < 0.001 within a 10,000 kb radius) were removed. When the data of representative SNPs were unavailable, we substituted them with proxy SNPs identifiable within LD (*r*^2^ > 0.8).

Subsequently, we conducted harmonisation to remove palindromic SNPs with an intermediate allele frequency. We then computed the F-statistic, retaining only instrumental variables with *F*-statistics (beta^2^/se^2^) > 10 to counteract the bias introduced by weak instruments [16].

### MR analysis

We used three MR methodologies to evaluate the influence of hypertension on epilepsy: the inverse-variance weighted (IVW) method, the weighted median (WM) method, and the MR-Egger method, due to their diverse assumptions regarding horizontal pleiotropy.

The IVW method, which calculates the weighted mean of the Wald ratio estimates of instrumental variables, typically offers the highest statistical power. Assuming the absence of horizontal pleiotropy, the IVW approach treats all extracted SNPs as valid instrumental variables. Therefore, we utilized this method as the primary analytical tool [17]. The WM method, on the other hand, assumes that at least 50% of the instrumental variables, sorted by weight, are valid, and that the horizontal pleiotropy is absent. In such scenarios, the WM method can yield a valid causal effect estimate [18]. The MR-Egger method does not necessitate the absence of horizontal pleiotropy. Instead, it allows for the possibility of unequal horizontal pleiotropy. This method requires the assumption that the impact of instrumental variables on the exposure is not associated with the direct effect of instrumental variables on the outcome. If this assumption holds true, the MR-Egger method can deliver causal effect estimates adjusted for bias, though its statistical power is generally lower compared to the IVW and WM methods [19]. In our analysis, a *P*-value < 0.05 was considered to denote statistical significance.

To ensure the robustness of our findings, we conducted a sensitivity analysis. Cochran's *Q* statistic was used to evaluate the heterogeneity, with a *P*-value < 0.05 indicating potential heterogeneity [20]. We also used the MR-Egger intercept method and the Mendelian randomization pleiotropy residuals and outliers (MR-PRESSO) test to screen for horizontal pleiotropy [17, 21]. A *P*-value < 0.05 suggests the possible presence of horizontal pleiotropy, which could undermine the results.

Scatter plots were generated to examine the potential causal relationships among hypothetical individuals. Leave-one-out analyses were performed to determine if the MR results overly rely on specific SNPs. Additionally, forest plots and funnel plots were created to visually inspect heterogeneity and horizontal pleiotropy.

All statistical analyses were performed using R version 4.3.0. The MR analysis was conducted using the TwoSampleMR (version 0.5.7) and MRPRESSO (version 1.0) softwares. Forest plots were generated using the "forestploter" function in R.

## Results

In this study, we designated hypertension as an exposure factor, yielding 18 SNPs with *F*-values ranging 30.59–81.71. When considering systolic blood pressure as the exposure factor, 379 SNPs were identified, with *F*-values ranging 29.82–627.55. Similarly, when using diastolic blood pressure as the exposure factor, 385 SNPs were identified, with *F*-values ranging 29.70–815.82. As per the selection criteria for instrumental variables, all SNPs exhibited *F*-values > 10, indicating the absence of weak instrument bias.

**Fig. 2:**
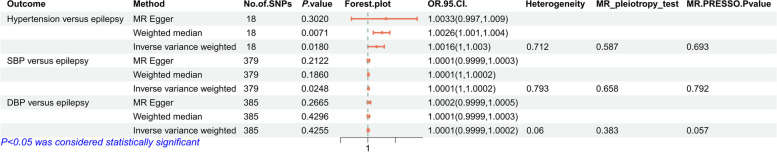
The relationship of hypertension, systolic blood pressure, or diastolic blood pressure with epilepsy

For the influence of hypertension on epilepsy, the IVW method indicated an association between hypertension and an elevated risk of epilepsy (odds ratio [OR] = 1.0016, 95% confidence interval [CI]: 1.000–1.003, *P* = 0.0180). This finding was confirmed by the WM method (OR = 1.0026, 95% CI: 1.001–1.004, *P* = 0.0071). When examining the impact of systolic blood pressure on epilepsy, the IVW analysis showed that increased systolic blood pressure could be linked to a higher risk of epilepsy onset (OR = 1.0001, 95% CI: 1.000–1.000, *P* = 0.0248). However, this association was not confirmed by the WM and the MR-Egger methods. For the effect of diastolic blood pressure on epilepsy, the IVW method revealed no significant causal relationship between diastolic blood pressure and epilepsy risk (OR = 1.0001, 95% CI: 1.000–1.000, *P* = 0.4255) (Fig. 2).

For sensitivity analyses, the *P*-values derived from Cochran's *Q* test were consistently > 0.05, indicating an absence of notable heterogeneity. The MR-Egger intercept did not exhibit significant deviation towards zero (*P* > 0.05), and the *P*-value for MR-PRESSO was also > 0.05. These results collectively suggest an absence of significant pleiotropy.

The leave-one-out analysis further demonstrated that the MR analysis results remained largely unchanged upon the removal of specific SNPs, indicating that the observed causal effect was not driven by a single instrumental variable. This was further corroborated by the funnel plots, which also displayed robust MR results. Taken together, these findings underscore the robustness of our data and suggest an absence of apparent bias, implying the credibility of our study outcomes.

## Discussion

In our research, we identified an association of hypertension, specifically increased systolic blood pressure, with a higher risk of epilepsy. However, no significant causal link was observed between increased diastolic blood pressure and seizure susceptibility. Although the OR value was close to 1, the statistical results should be interpreted with caution. We acknowledge the relatively small effect size indicated by the OR, suggesting that while hypertension may contribute to epilepsy risk, this influence is likely subtle and may potentially interact with other factors. While we observed a modest OR for systolic blood pressure in relation to epilepsy risk, this might be due to the potential influence of a dilution effect. This phenomenon, often encountered in epidemiological studies, occurs when the variability within the exposure group reduces the apparent strength of the association. In our study, the heterogeneous nature of the hypertensive population in terms of severity, duration, and treatment adherence could have contributed to a dilution of the observed effect. Thus, while the systolic blood pressure showed an association with epilepsy, the diversity of characteristics and management of hypertension among the study participants may have led to an underestimation of the true effect size. Future studies with more homogenous and well-defined hypertension cohorts are needed. In conclusion, the results of this study showed the potential impact of hypertension on epileptogenesis, indicating the importance of enhanced blood pressure monitoring and management to mitigate epilepsy recurrence risk in patients.

Our research lends support to the hypothesis that hypertension could be a potential risk factor for epilepsy. Our findings augment the hypothesis that hypertension may contribute to the epilepsy etiology, as evidenced by a notable correlation between these conditions. However, it is crucial to note that the observed correlation, while statistically significant, is a weak association. This necessitates cautious interpretation to avoid overestimation of the impact of hypertension on epilepsy. In addition, the aim of this study was to define correlation, rather than mechanism [22]. The RAS in the brain, implicated in diverse neurological functions, is emerging as a theoretical mechanism potentially linking hypertension to epilepsy [23]. However, our study did not directly analyze the involvement of RAS in epileptogenesis. Therefore, discussions regarding the role of RAS remain hypothesized. In the future, studies are needed to clarify the role of the RAS pathway in the hypertension-epilepsy interplay. Emerging evidence suggests a pivotal role for neuroinflammation in epilepsy pathogenesis [24]. Seizures have been associated with a significant increase in angiotensin II (Ang II) expression in activated microglia. Ang II and its AT1 receptor can amplify neuroinflammatory responses, leading to neurodegeneration [25]. Therefore, inhibition of the AT1 receptor may reduce levels of inflammation and oxidative stress. In epilepsy, RAS inhibitors could serve as anti-inflammatory and neuroprotective agents. Angiotensin-converting enzyme inhibitors (ACEI), for instance, has been reported to mitigate neuroinflammation and prevent neuronal loss in the hippocampus during epilepsy [26]. Furthermore, the role of the RAS in the brain, particularly in the context of epilepsy, warrants deeper exploration. The elevation of Ang II during seizures, and its interaction with AT1 receptors, may amplify neuroinflammatory responses, leading to increased neuronal excitability and susceptibility to seizures. Inhibiting AT1 receptors could, therefore, offer a dual benefit of reducing inflammation and attenuating the epilepsy risk.

Our findings suggest that an increase in systolic blood pressure may be associated with an elevated epilepsy risk. This association could be due to the potential vascular damage, including arteriosclerosis and vasoconstriction, caused by increased systolic pressure, subsequently affecting the cerebral blood flow and neuronal activity. Vascular damage could also disrupt the blood-brain barrier, potentially disturbing the neurotransmitter balance and increasing epilepsy risk [27]. However, we found no significant causal relationship between diastolic blood pressure and epilepsy risk, possibly due to the lesser impact of diastolic pressure changes on cerebral blood flow.

Our results were consistent with the study by Wilner et al., which identified hypertension as the most common comorbidity in a group of epilepsy patients aged over 19 [28]. However, we also found that the systolic blood pressure increase may be associated with elevated epilepsy risk, while the diastolic blood pressure did not show the same correlation. This suggests that different types of hypertension may have different impacts on epilepsy risk. This finding warrants validation in larger cohorts and further biological studies to elucidate how systolic and diastolic blood pressures influence the epilepsy risk.

Despite providing new insights into the relationship of hypertension, systolic and diastolic blood pressure with the epilepsy risk, our study has limitations. First, The MR approach, which relies on genetic variation as instrumental variables, may cause weak instrumental variable bias, although we attempted to mitigate the bias by selecting SNPs with *F*-values > 10. Second, our reliance on publicly available datasets may limit our control over potential confounders. For instance, we did not have information on the lifestyles, dietary habits, and physical activity levels of patients, all of which could influence the association between hypertension and epilepsy risk. Third, we did not directly measure neuroinflammation or blood–brain barrier disruption. Therefore, it remains unclear how systolic and diastolic blood pressures influence the epilepsy risk. Future research may necessitate more direct measurement methods. Fourth, the sample size was relatively small in our study, as only 3810 cases from the UK Biobank were included. The small sample size may have constrained the statistical power and the robustness of our results. Lastly, our study primarily focused on European populations, which limits the generalization of the findings to other ethnic groups.

Future research should focus on the underlying mechanisms of the association between hypertension, particularly systolic blood pressure, and the risk of epilepsy. The roles of the RAS and neuroinflammation in the pathogenesis of epilepsy in the context of hypertension warrant further investigation. Additionally, larger and diverse cohorts, including different ethnic backgrounds and age groups, should be included to validate our findings and ensure generalizability. Longitudinal studies with hypertension monitoring over time could provide insights into the temporal relationship between hypertension and epilepsy. Lifestyle factors, such as diet, physical activity, and stress levels, should be taken into account to remove potential confounders. Furthermore, interventional studies assessing the impact of strategies aimed at controlling hypertension, such as antihypertensive medication or lifestyle modifications, on the incidence or severity of epilepsy could provide valuable insights.

If validated, our findings could have significant clinical implications for epilepsy prevention and treatment. For instance, if hypertension does indeed increase the epilepsy risk, managing hypertension could become a crucial strategy for epilepsy prevention. Moreover, if systolic and diastolic blood pressures differentially impact the epilepsy risk, hypertension treatment strategies could be more specific. For example, more emphasis could be placed on controlling systolic rather than the overall blood pressure. However, these hypotheses require further validation.

It is also important to highlight the small effect size and the weak correlation observed in this study, calling for cautions against overinterpreting the data as definitive evidence of a strong causal relationship. Instead, these findings should be viewed as an initial step to understand a complex and possibly multifaceted relationship between hypertension and epilepsy.

## Conclusions

In conclusion, our MR study suggests that hypertension, particularly systolic blood pressure, is potentially associated with an increased risk of epilepsy. Further investigations are needed to elucidate the potential mechanisms underlying the causal relationship between hypertension and epilepsy.

## Data Availability

The datasets analysed during the current study are available in the IEU OpenGWAS (https://gwas.mrcieu.ac.uk/).

## Abbreviations

ACEI: Angiotensin-converting enzyme inhibitors
ANG II: Angiotensin II
AT1: Angiotensin II receptor type 1
BMI: Body mass index
CI: Confidence interval
DNA: Deoxyribonucleic acid
GWAS: Genome-wide association studies
HDL: High-density lipoprotein
ILAE: International League Against Epilepsy
IVW: Inverse variance weighted
LD: Linkage disequilibrium
LDL: Low-density lipoprotein
MR: Mendelian randomization
MR-PRESSO: Mendelian randomization pleiotropy residual sum and outlier
OR: Odds ratio
QTL: Quantitative trait loci
RAS: Renin-angiotensin system
RCT: Randomized controlled trial
RNA: Ribonucleic acid
SNP: Single nucleotide polymorphism
WM: Weighted median
